# Gd–Pt–In *In Situ* Composite Exhibiting Enhanced Relative Cooling Power and Broad Magnetocaloric Effect

**DOI:** 10.1021/acsomega.5c06638

**Published:** 2025-11-13

**Authors:** Aline A. de Freitas, Jessica K. P. França, Walajhone O. Pereira, Alan S. de Menezes, Adenilson O. dos Santos, Luzeli M. da Silva

**Affiliations:** † Center for Sciences of Imperatriz, 37892 Federal University of Maranhão (UFMA), Imperatriz, MA 65900-410, Brazil; ‡ Department of Physics, Federal University of Maranhão (UFMA), Campus Bacanga, São Luís, MA 65080-805, Brazil

## Abstract

In this study, a Gd–Pt–In composite was synthesized by arc melting. Powder X-ray diffraction, scanning electron microscopy (SEM), and energy-dispersive X-ray spectroscopy (EDS) analyses confirmed the composite composition formed by a matrix phase of GdPtIn, comprising 76% of the sample, and a dispersed phase (21%) formed by GdPt. Magnetic and specific heat measurements revealed two ferromagnetic transitions in the composite at 67 and 63.5 K. These successive transitions provide a very broad curve for both the magnetic entropy change (−Δ*S*_M_) and adiabatic temperature change (Δ*T*_ad_) magnetocaloric parameters, generating a wide temperature range with a significant magnetocaloric effect (MCE) and a substantial relative cooling power (RCP). For a magnetic field change (Δ*H*) of 50 kOe, the maximum magnetic entropy change (−Δ*S*_M_^max^) reached 6 J/kg K, with a corresponding RCP value of 368 J/kg. The excellent magnetocaloric properties of the Gd–Pt–In composite position it as a promising candidate for low-temperature magnetic refrigeration applications. Furthermore, these findings offer valuable insights for the ongoing search for effective refrigerant materials, particularly those exhibiting multiple successive magnetic phase transitions.

## Graphical abstract

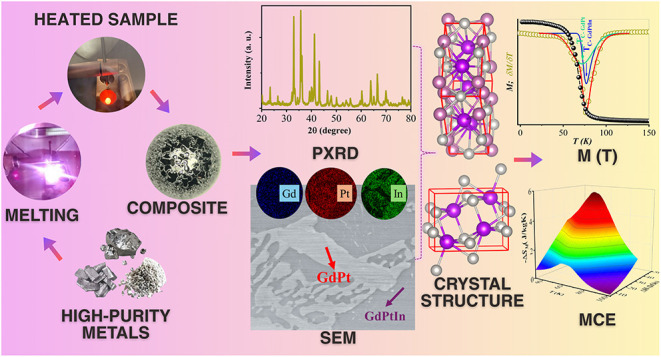

## 1 Introduction

In the coming decades, society will experience a significant increase in demand for renewable energy, driven by the urgent need to reduce carbon emissions associated with fossil fuel combustion. In this context, green hydrogen will play a pivotal role in the transition to a carbon-neutral society.^1–3^ Global hydrogen demand is projected to increase 5-fold by 2050, reaching approximately 550 million tons.^4^ Thus, reducing the energy and economic costs of hydrogen liquefaction becomes crucial. In this context, alternative refrigeration processes, specifically technologies based on the i-caloric effect, emerge as a promising and important solution to support advancements in fields like conventional hydrogen liquefaction technologies.^5–7^

The i-caloric effects describe the reversible temperature change (under adiabatic conditions) or an entropy change (under isothermal conditions) that a solid material undergoes when an external stimulus (magnetic field, electric field, or mechanical pressure) is applied or removed.^8,9^ Specifically for magnetic materials, the magnetocaloric effect (MCE) is triggered by applying an external magnetic field near the magnetic phase transition temperature of the material. Despite extensive research on materials exhibiting MCE across various temperature ranges, this field remains a hotbed of scientific inquiry due to its great potential for technological applications. Significant effort has been invested in developing materials with strong magnetocaloric performance over a broad temperature range by using only moderate magnetic fields. Achieving optimal performance requires materials with a high and near-constant magnetic entropy change (−Δ*S*_M_) across the entering operating temperature range (a table-like profile), which is essential for reaching Ericsson cycle efficiency.^10,11^ Crucially, for successful implementation in magnetic refrigeration, researchers must effectively control critical parameters, including hysteresis losses, low thermal conductivity, and the narrow range of the material magnetic phase transition.^12–14^

Recent studies have identified promising materials exhibiting table-like behavior, including those with multiple magnetic phase transitions, multiphase systems, and composites.^15–19^ To achieve the desired table-like MCE, enhancing the properties of magnetic composites is imperative.^20,21^ Broadening Δ*S*_M_ curves expands the working temperature range, thereby increasing cooling capacity.^22,23^ However, attaining these features in a single material remains challenging. Researchers have thus focused on developing composites that combine magnetocaloric materials with tailored Curie temperatures (*T*_C_) to overcome these limitations.

While some multilayer or powder composites (e.g., Eu_8_Ga_16_Ge_30_/EuO,^16^ (Tm/Dy/Tb)­Mn_2_Si_2_-HoCoSi,^24^ and Fe_87_Zr_6_B_6_Cu_1_/Fe_90_Zr_8_B_2_ ^18^) exhibit table-like behavior, they face drawbacks such as low thermal conductivity, porosity, and structural defects that hinder performance.^25^ In contrast, in situ composites, formed by controlled-phase integration, emerge as promising alternatives to address these issues.

Gadolinium (Gd) stands out due to its good magnetocaloric effect (MCE) near room temperature. However, its low mechanical strength and corrosion susceptibility pose challenges for practical applications.^20,26^ To mitigate these, researchers have explored alloying Gd with elements like Cu, Ni, Ga, In, Ag, Au, and Pt.^27–30^ Studies show that trace additions can tune the Curie temperature of gadolinium and enhance its magnetocaloric properties compared to pure Gd.^31–33^

The combination of Gd, Pt, and In yields compounds with diverse compositions and strategically relevant magnetic transitions. For instance, coupling GdPtIn (68 K)^34^ and GdPt (66 K)^35^ phases could enable composites with successive transitions in a critical temperature range between nitrogen (77 K) and hydrogen (20 K) liquefaction points.^36^ This opens avenues for developing advanced systems with optimized magnetocaloric properties.

In this study, we propose synthesizing a multiphase intermetallic composite of Gd, Pt, and In via arc melting. The magnetocaloric properties were investigated, revealing a broad −Δ*S*_M_ peak.

## 2 Experimental Section

A Gd–Pt–In sample with a nominal composition of 33 wt % Gd, 42 wt % Pt, and 24 wt % In (total mass ≈ 1 g) was prepared by arc melting using Gd = 99.95%, Pt = 99.99%, and In = 99.999% constituent elements (all supplied by Sigma-Aldrich). The pure metals were melted in a water-cooled copper crucible under a high-purity argon atmosphere. The arc-melted button was turned and remelted three times to ensure homogeneity. The sample was then sealed directly into the fused silica ampule under an ultrapure argon atmosphere (∼900 mbar) and annealed at 800 °C for 7 days, followed by cooling-water quenching. After annealing, no detectable contamination or reaction between the sample and the fused silica ampule was observed.

Powder X-ray diffraction (PXRD) measurements were performed to identify the crystalline phase present in the sample. The diffraction pattern was obtained using a Panalytical Empyrean diffractometer with Cu Kα radiation (λ = 1.5418 Å), operating at 40 kV and 40 mA. Diffractograms were collected at room temperature over a 2θ range of 20–100°, with a step size of 0.02° and an acquisition time of 2 s per step. Structural analysis was conducted via the Rietveld method^37^ using the GSAS-EXPGUI software.^38,39^

The crystalline phase fractions within the composite were determined using Rietveld refinement with GSAS (General Structure Analysis System) software. This method fits a calculated diffraction pattern to experimental data using the least-squares method. The scale factors (*S*_i_) are among the crystallographic parameters adjusted during the refinement. The mass fraction (*W*_i_) of each phase was then calculated from these scale factors, along with the number of formula units per unit cell (*Z*_i_), the molar mass (*M*_i_), and the unit cell volume (*V*_i_), according to the following equation^40^

1 $$W_{i}=\frac{S_{i}Z_{i}M_{i}/V_{i}}{\sum_{j}S_{j}Z_{j}M_{j}/V_{j}}$$

where the sum in the denominator includes all phases considered in the refinement. The parameters *Z*_i_, *M*_i_, and *V*_i_ were derived from the structural models of each phase. Specifically, *V* was calculated directly from the lattice parameters refined in GSAS. The resulting mass fractions, expressed as percentages, are termed ″phase percentage” throughout this study.

Morphological characterization was performed using a Zeiss Evo HD scanning electron microscope (SEM), equipped with a secondary electron (SE) detector. Energy-dispersive X-ray spectroscopy (EDS), using a Bruker XFlash 410 M detector, was also employed to identify the chemical elements present in the sample. Prior to morphological analysis, the sample surface was manually polished in a progressive sequence using wet SiC sandpaper with grit sizes of 600, 800, 1200, and 2400 for 2–3 min, starting with the coarsest and progressing to the finest, to obtain a flat surface free of scratches and deep marks. Subsequently, the sample was finely polished using diamond pastes with particle sizes ranging from 7 to 0.25 μm. The polished sample was mounted directly on double-sided conductive carbon tape without a metal coating.

DC magnetization measurements were carried out using a Quantum Design Inc. MPMS SQUID magnetometer in the temperature range of 2–120 K under applied magnetic fields up to 50 kOe. The temperature dependence of magnetization, *M*(*T*), was obtained following a field-cooled warming (FCW) protocol. In this protocol, the sample was cooled to room temperature under an applied magnetic field. After the temperature was stabilized at 2 K, the *M*(*T*) curve was recorded as a function of increasing temperature. Heat capacity measurements were performed on a Quantum Design Inc. Dynacool PPMS (Physical Properties Measurement System) was used at applied magnetic fields of zero, 20, and 50 kOe within the 2–120 K temperature range. As confirmed by the SEM analysis, the composite exhibits a uniform microstructure; therefore, all measured quantities were normalized to the total sample mass.

## 3 Results and Discussion

### 3.1 PXRD Analysis

The powder X-ray diffraction pattern of the Gd–Pt–In sample is presented in Figure 1a, where the experimental data (circles) are compared with the Rietveld-refined calculated profile (red line), along with the corresponding difference curve (blue line). The analysis indicates that the primary phase, GdPtIn, constitutes approximately 76% of the sample and crystallizes in a hexagonal Fe_2_P-type structure (space group *P*6̅2*m*).^41^ A second phase, GdPt, accounts for 21% of the material and adopts an orthorhombic FeB-type structure (space group *Pnma*).^42^ Additionally, peaks corresponding to Gd_2_O_3_ (indicated by asterisks in the diffractogram), with a cubic structure (space group *Ia*3̅), account for 3% of the sample.^43^ It is important to note that the peak at 2θ ≈ 39.32° exhibits a significantly higher intensity compared with the most intense peak for this phase, located at 2θ ≈ 28.44°. This occurs due to the preferential orientation of the (332) crystallographic reflection. Additionally, a minor preferential orientation is also observed in the peak at 2θ ≈ 41.2°, associated with the (300) reflection of the GdPtIn phase. The multiphase nature of the sample poses a considerable challenge to achieving a fine powder with a uniform grain size. Such grain size inhomogeneity typically arises from differences in the hardness of the constituent phases, which can lead to uneven grinding and, consequently, the development of preferential orientations in the diffraction pattern. Figure 1b,c displays the individual diffraction patterns of the GdPtIn and GdPt phase, while Figure 1d,e illustrates their respective unit cells used in the Rietveld refinement. The powder X-ray diffraction analysis evidence that the Gd–Pt–In is a composite material consisting of GdPtIn and GdPt intermetallic compounds and a small amount of Gd oxide.

**Figure 1:**
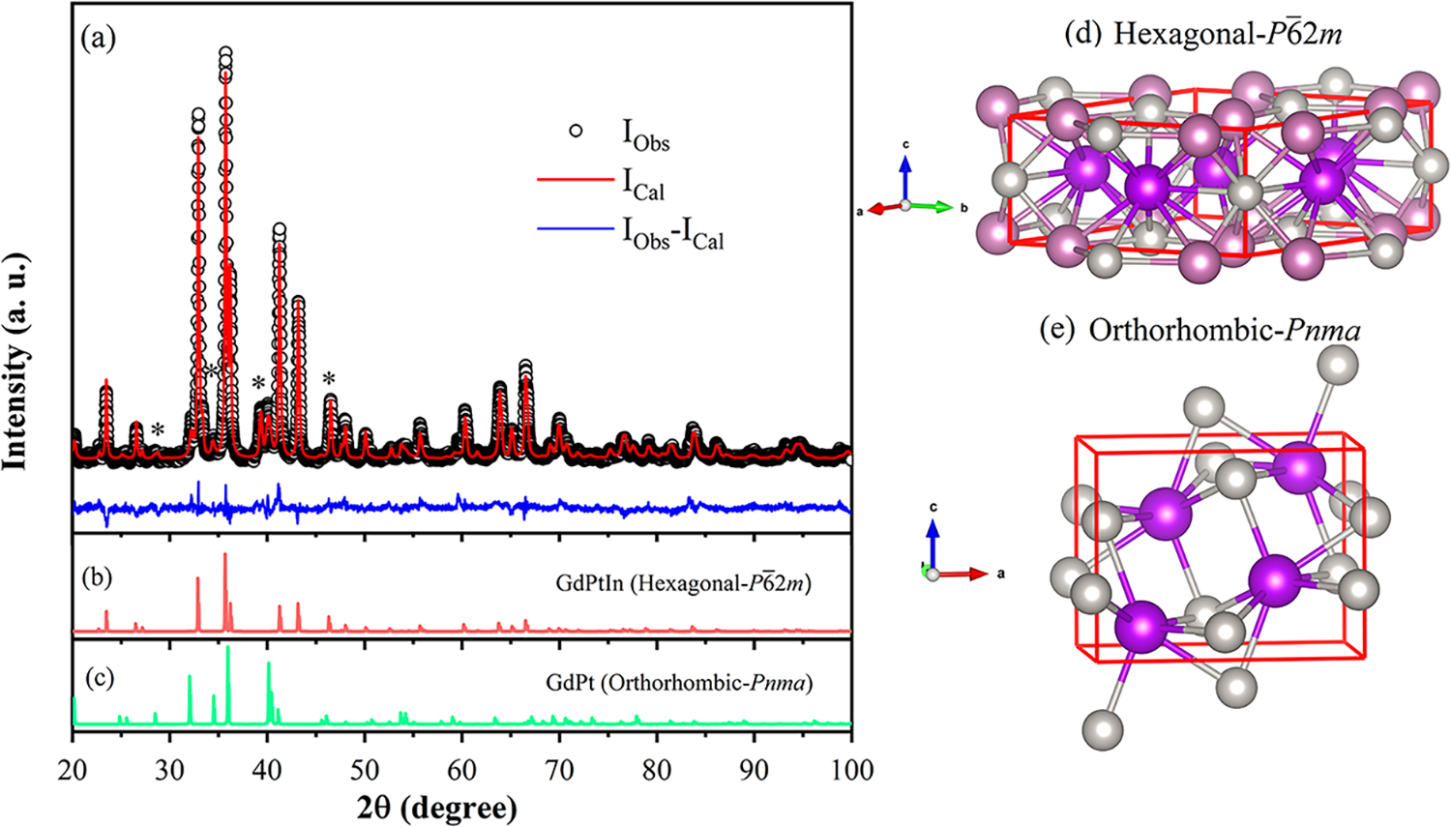
(a) Powder X-ray diffraction pattern of the Gd–Pt–In sample, displaying experimental (black circles) and calculated (red line) profiles with the difference plot in blue. The asterisks correspond to the Gd oxide peaks. (b, c) Individual diffraction patterns for each identified phase. (d, e) Representations of the Hexagonal and orthorhombic unit cells corresponding to the intermetallic compounds GdPtIn and GdPt, respectively.

Powder X-ray diffraction analysis unambiguously identifies the composite system comprising GdPtIn, GdPt, and Gd_2_O_3_ as crystalline phases. Table 1 summarizes their lattice parameters, unit cell volume, and phase fractions, along with reliability factors (*R*_p_, *R*_wp_, and *S*). It is worth noting that these reliability factors are somewhat elevated compared to the ideal values expected from an optimized refinement. This deviation can likely be attributed to preferred orientation effects present in the diffractogram and to the inherent challenges of refining a composite material with three coexisting phases. Notably, the refined structural parameters of the Gd–Pt–In composite are in good agreement with those reported in the literature for the compounds GdPtIn,^41^ GdPt,^42^ and Gd_2_O_3_.^43^

**Table 1:** Lattice Parameters, Unit Cell Volume, Phase Fractions of the Gd–Pt–In Composite, and Reliability Factors from the Rietveld Refinement^a^

	space group	structure	*a* (Å)	*b* (Å)	*c* (Å)	*V* (Å^3^)	phase fraction	ref
GdPtIn	*P*6̅2*m*	hexagonal	7.575(3)		3.902(4)	193.90(6)	76%	this study
			7.573		3.917	194.6		^41^
GdPt	*Pnma*	orthorhombic	7.085(3)	4.465(6)	5.593(5)	176.91(8)	21%	this study
			7.088	4.499	5.591	178.31		^42^
Gd_2_O3	*Ia*3̅	cubic	10.735(5)			1237.27(2)	3%	this study
			10.823			1267.78		^43^
reliable factors			*R*_wp_ = 12%		*S* = 1.59		*R*_p_ = 9%	

a For comparison, the structural parameters of GdPtIn, GdPt, and Gd_2_O_3_ reported in the literature are also included.

### 3.2 SEM and EDS Analysis

The photomicrograph of the Gd–Pt–In composite, Figure 2a,b, revealed a homogeneous microstructure containing three distinct phases: a matrix, a dispersed phase exhibiting directional growth, and a minor third phase. The predominant dark gray area (indicated by a red arrow) constitutes the matrix phase, accounting for the majority of the sample volume. A substantial amount of a dispersed, light gray phase (orange arrow) is noticeably dispersed within this matrix. Additionally, some black regions (white arrows) are also present in the sample. The small white bright spots in Figure 2a are identified as residual polishing debris. Further insights are provided by the compositional maps in Figure 2b–d, which demonstrate a uniform distribution of the Gd. In contrast, the light gray regions observed in the SEM image indicate an absence of indium. The dark regions lack both Pt and In, suggesting the presence of Gd oxide. Energy-dispersive X-ray spectroscopy spectra, shown in Figure 2e,f, corroborate these findings: the matrix exhibits the presence of Gd, Pt, and In elements, whereas the dispersed phase solely contains Gd and Pt. Collectively, a comparative analysis of the SEM micrographs (Figure 2a), EDS spectra (Figure 2e,f), and PXRD data (Figure 1a) confirms that the dark gray matrix is the GdPtIn phase and the dispersed phase is GdPt.

**Figure 2:**
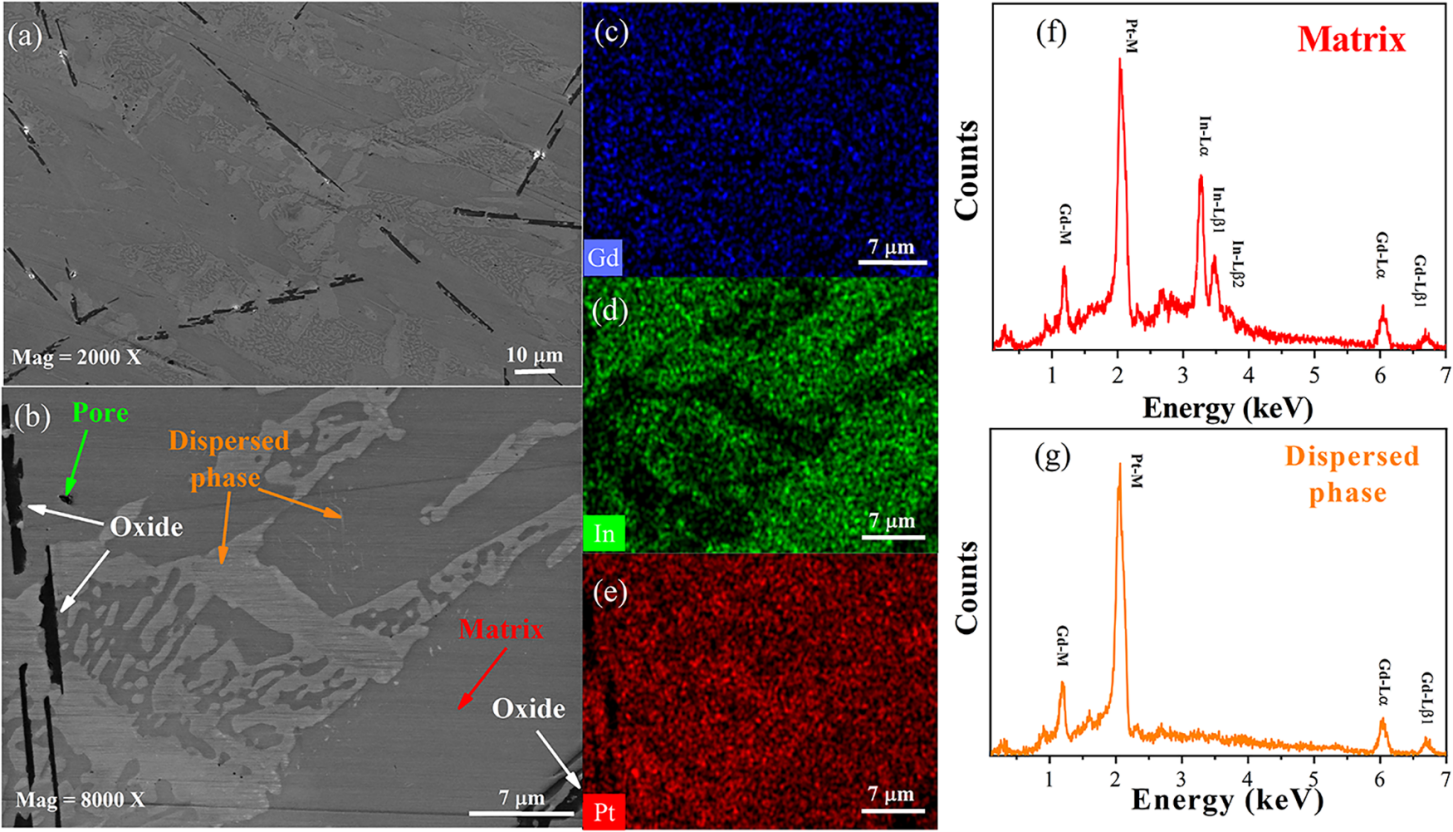
Microstructural and elemental studies of the Gd–Pt–In sample by SEM and EDS techniques. Photomicrograph at (a) 2000× and (b) 8000× magnification. (c–e) Elemental distribution mapping by EDS (area scanning analysis). (f, g) EDS spectra.

### 3.3 Magnetic Properties

Figure 3a shows the FCW magnetization of the Gd–Pt–In composite as a function of temperature, recorded from 2 to 150 K with an applied magnetic field of 500 Oe. The magnetization curve indicates a paramagnetic (PM) behavior above 80 K. Below this temperature, the material undergoes a notable magnetic transition, characterized by a sharp increase in magnetization. To better resolve these transitions, the derivative of magnetization with respect to temperature is shown in the inset of Figure 3a. This derivative reveals two overlapping minima, which upon deconvolution analysis confirm the presence of two distinct ferromagnetic (FM) transitions. The first minimum, designated as *T*_C1_ and located around 67 ± 0.3 K, is indicative of the PM to FM transition associated with the GdPtIn phase. The second minimum, *T*_C2_ at approximately 64 ± 0.5 K, corresponds to the PM to FM transition of the GdPt phase. Notably, the determined *T*_C1_ value aligns well with the Curie temperature values reported in the literature for the GdPtIn compound.^34,44^ However, the observed *T*_C2_ is slightly reduced compared to the 66 K literature value for the GdPt compound.^35^ Figure 3b displays the isothermal magnetization curves measured at 2 and 10 K. For both curves, the magnetization increases progressively and reaches saturation for magnetic fields above 20 kOe, confirming the ferromagnetic behavior. The experimental saturation magnetization was determined to be 85.9 emu/g (6.69 μ_B_/Gd ion), which is in good agreement with the theoretical value of 87 emu/g estimated using the model proposed by Park et al.,^45^ taking into account the mass fractions of GdPt and GdPtIn obtained from the PXRD data analysis. In the theoretical calculation of the saturation magnetization per unit formula for each phase, Gd^3+^ ions (*J* = 7/2) were assumed to be in a perfectly aligned magnetic state.

**Figure 3:**
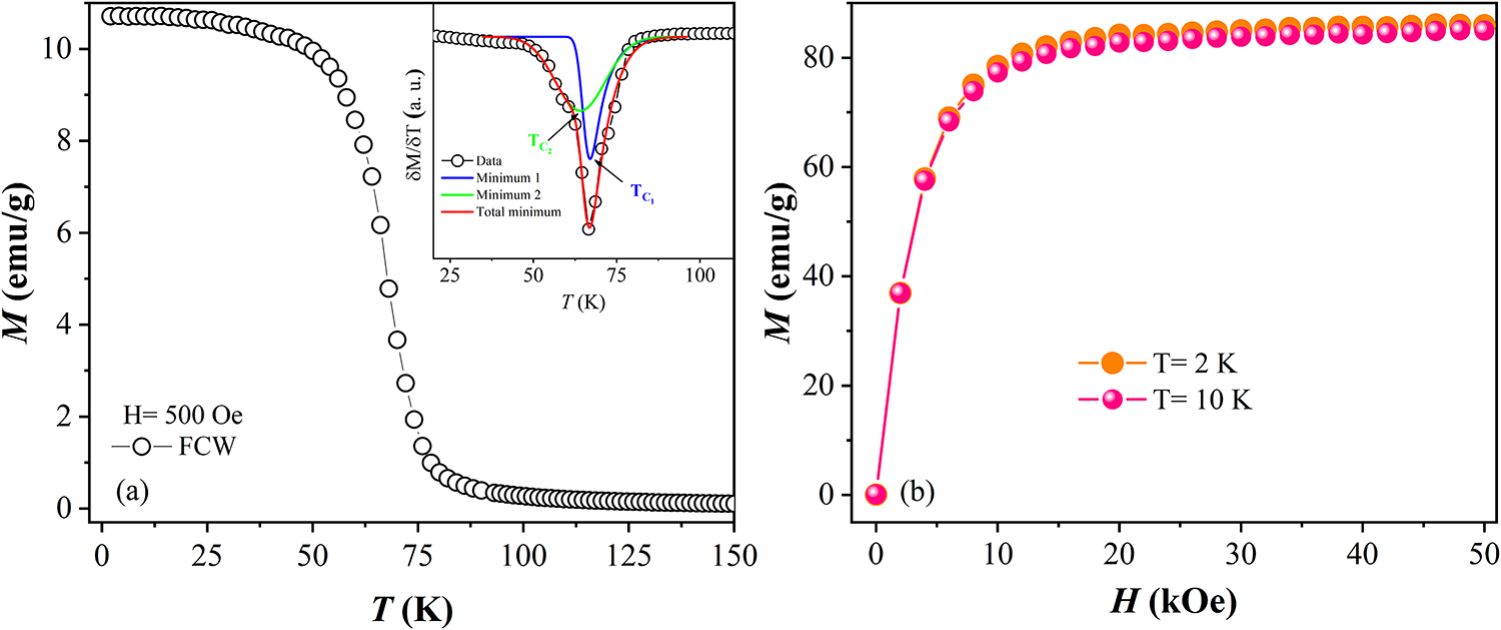
(a) Magnetization as a function of temperature measured in the FCW mode for the Gd–Pt–In composite. In the inset, the magnetization derivative shows the minima corresponding to the ferromagnetic transitions at *T*_C1_ and *T*_C2_, together with its deconvolution using two Gaussian functions. (b) Isothermal magnetization curves at *T* = 2 and 10 K.

Figure 4 presents the isothermal magnetization curves of the Gd–Pt–In composite recorded across an applied magnetic field range of 0 to 50 kOe and at temperatures spanning from 26 to 104 K. At lower temperatures, the magnetization behavior is consistent with the ferromagnetic nature of the composite. In contrast, for temperatures exceeding 80 K, the curves no longer exhibit saturation, as expected, since all constituent phases are in the paramagnetic state above their respective Curie temperatures.

**Figure 4:**
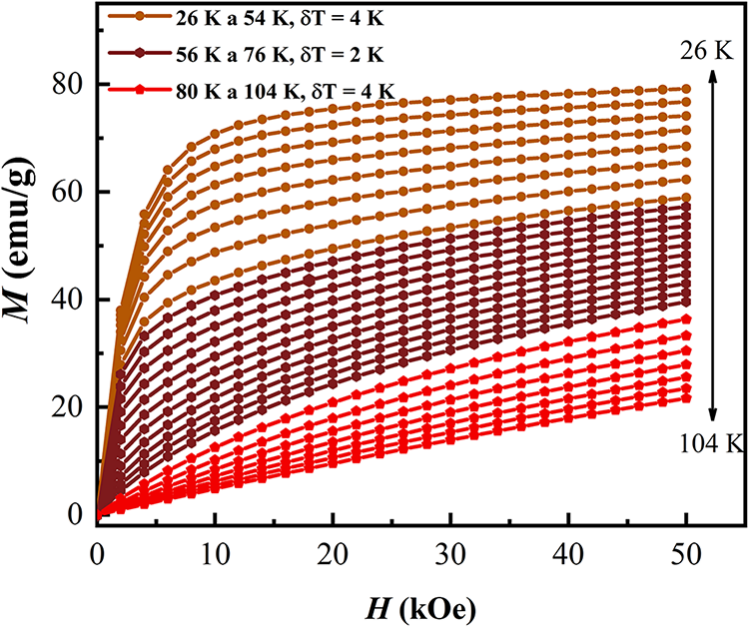
Isothermal magnetization of the Gd–Pt–In composite as a function of the magnetic field for the temperature range from 2 to 59 K.

Figure 5a illustrates the heat capacity (*C*_p_) curves as a function of temperature at zero magnetic field, 20 and 50 kOe. The zero-field curve shows two distinct contributions, marked by light-pink and light-yellow bands, which correspond to the ferromagnetic transitions at *T*_C1_ and *T*_C2_. Rather than sharp λ-type peaks, these contributions appear as broadened discontinuities. The onset and offset temperatures for these transitions were 70 and 64 K for *T*_C1_, and 60 and 56 K for *T*_C2_. The temperature shift observed for the *T*_C2_ transition, when compared with magnetization data, can be attributed to several factors. These include short-range magnetic fluctuations, the intrinsic nature of the phase transitions, and the overlap of contributions from the two distinct phases.

**Figure 5:**
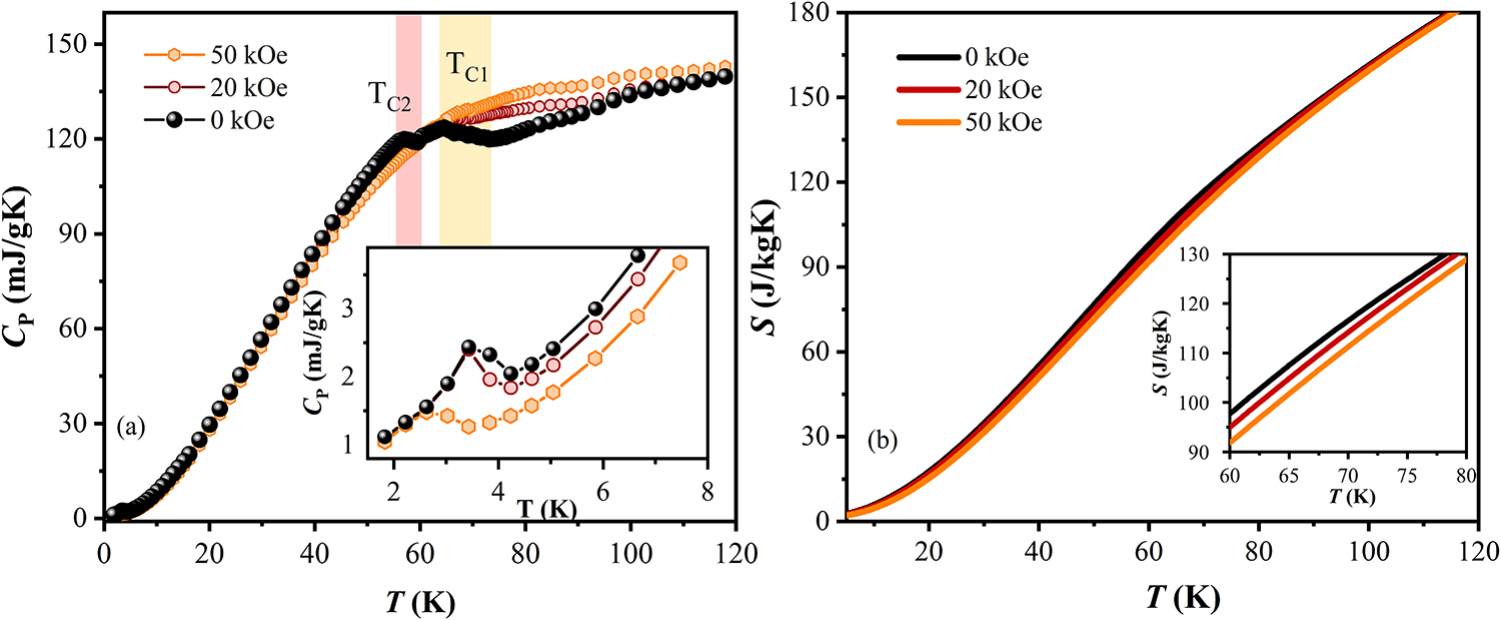
(a) Heat capacity (*C*_P_) as a function of temperature, measured at zero field, 20, and 50 kOe. In the inset, an enlarged view of the region below 6 K. (b) Entropy (*S*) as a function of temperature with an enlarged view of the temperature interval from 60 to 80 K (inset).

Applying a magnetic field significantly decreases the contribution of these transitions, leading to a broadening of the *C*_P_ curve. In addition to the ferromagnetic contributions, a low-intensity, well-defined λ-type peak is observed in the heat capacity of the Gd–Pt–In composite, as detailed in the inset of Figure 5a. This peak, initially located at around ∼3.6 K, shifts to ∼3.1 K with no significant change in its shape. The peak’s shape and its behavior under a 50 kOe magnetic field suggest an AFM transition, which is likely attributed to the Gd oxide. Previous studies have documented an antiferromagnetic transition at 3.5 K for biphasic (cubic and monoclinic) Gd_2_O_3_ nanoparticles.^46^ Furthermore, low-temperature antiferromagnetic interactions have been observed for cubic nanorod and nanosheet forms of Gd_2_O_3_.^47^

Figure 5b presents the entropy curves, *S*(*T*), as a function of temperature, derived from the heat capacity data shown in Figure 5a. These curves were calculated for measurements performed at zero, 20, and 50 kOe using the relation^48^

2 $$S{\left(T\right)}_{H}=\int_{0}^{T}\frac{C_{p}{\left(T\right)}_{H}}{T}dT$$

As shown in the inset of Figure 5b, the magnetic field reduces the entropy around the magnetic transitions, evident from the separation of the 20 kOe and 50 kOe curves from the zero-field curve.

The isothermal magnetic entropy change was calculated by using two distinct approaches. First, it was derived from the isothermal magnetization data, presented in Figure 4, through the integration of Maxwell’s relation,^48^ as expressed in eq 3

3 $$\Delta S_{M}{\left(T\right)}_{\Delta H}=\int_{H_{i}}^{H_{f}}{\left(\frac{\partial M{\left(H\right)}_{T}}{\partial T}\right)}_{H}dH$$

Second, it was obtained from specific heat data by subtracting the entropy curves,^48^ as outlined in eq 4

4 $$\Delta S_{M}(T{)}_{\Delta H}={\left[S(T{)}_{H_{f}}-S(T{)}_{H_{i}}\right]}_{T}$$

Figure 6a illustrates the −Δ*S*_M_ as a function of temperature and magnetic field change (Δ*H*), calculated from isothermal magnetization data using eq 3, for Δ*H* = 10, 20, 30, 40, and 50 kOe. These results are compared with those obtained from specific heat measurements, calculated using eq 4, for Δ*H* = 20 and 50 kOe. The −Δ*S*_M_ curves derived from *C*_P_ closely match those obtained from magnetization, exhibiting an asymmetric and broad profile with a maximum value of around 65 K. This broadened shape results from the overlapping magnetic contributions associated with the ferromagnetic ordering of GdPtIn and GdPt at their respective transition temperatures, *T*_C1_ and *T*_C2_. The broadening becomes more pronounced as the applied magnetic field increases. A broadened and nearly constant profile for −Δ*S*_M_ is beneficial for magnetic refrigeration applications and was successfully achieved in the Gd–Pt–In composite.

**Figure 6:**
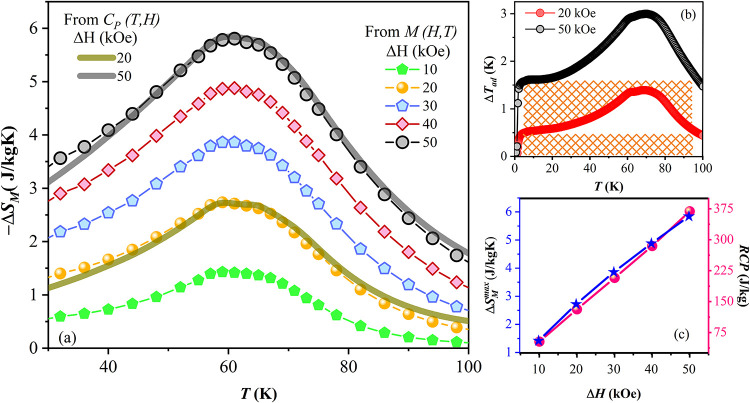
(a) Temperature dependence of the −Δ*S*_M_ under different magnetic field changes, comparing values obtained from magnetization and specific heat measurements. (b) Adiabatic temperature change versus temperature for Δ*H* = 20 and 50 kOe, hatched area indicates the constant value of Δ*T*_ad_ over a wide temperature range. (c) Field dependence of −Δ*S*_M_^max^ (left axis) and RCP (right axis) for the Gd–Pt–In composite.

The adiabatic temperature variation (Δ*T*_ad_) is another crucial parameter for assessing the magnetocaloric performance. It can be represented as the isentropic temperature difference between the entropy curves measured in the initial (*H*_i_) and final (*H*_f_) magnetic fields, denoted as *S*(*T*)_*H*_i__ and *S*(*T*)_*H*_f__, respectively. The Δ*T*_ad_ value can be calculated from these entropy curves using the following expression.^48^

5 $$\Delta T_{\mathrm{ad}}(T{)}_{\Delta H}={\left[T(S{)}_{H_{f}}-T(S{)}_{H_{i}}\right]}_{S}$$

Figure 6b shows the Δ*T*_ad_ as a function of the temperature. The maximum Δ*T*_ad_ value reaches 1.4 and 3.0 K for Δ*H* = 20 and 50 kOe, respectively. Notably, Δ*T*_ad_ exhibits a plateau in the temperature range 5 to 95 K, with its intensity increasing proportionally to Δ*H*. Within this temperature, Δ*T*_ad_ remains constant at values of 0.5 and 1.6 K for Δ*H* = 20 and 50 kOe, respectively. Figure 6c displays the dependence of the maximum magnetic entropy change (−Δ*S*_M_^max^) as a function of Δ*H* for the Gd–Pt–In composite. The −Δ*S*_M_^max^ values increase with the applied field and show no saturation trend for field variations up to 50 kOe, reaching a maximum value of 6 J/kg K. The relative cooling power (RCP), a parameter used to quantify the heat transferred between the hot and cold reservoirs in an ideal refrigeration cycle, was estimated using the relation presented in eq 6:^49^

6 $$\mathrm{RCP}=\left|{-\Delta S}_{M}^{\max}\right|.\delta T_{\mathrm{FWHM}}$$

where δ*T*_FWHM_ represents the full width at half-maximum of the −Δ*S*_M_ peak. The *RCP*, presented in Figure 6c, increases continuously with the applied magnetic field, mirroring the behavior of −Δ*S*_M_^max^. It increases continuously, with no saturation observed for magnetic field variations up to 50 kOe, where the composite achieves an RCP of 368 J/kg.

Table 2 compiles the key magnetocaloric parameters, including the maximum magnetic entropy change, the relative cooling power, and the full width at half-maximum of the −Δ*S*_M_ peak. These values are presented alongside the ordering temperatures and applied magnetic field changes for the Gd–Pt–In composite, as well as for other Gd-based materials reported in the literature, for comparative purposes. Our analysis reveals that the Gd–Pt–In composite exhibits −Δ*S*_M_^max^ comparable to those of other Gd-based materials documented in the literature in the same temperature range. These findings confirm an appreciable magnetocaloric effect, making the Gd–Pt–In composite a promising candidate for low-temperature magnetic refrigeration applications.

**Table 2:** Magnetic Transition Temperature (*T*_ord_) and Magnetocaloric Parameters (−Δ*S*_M_^max^, δ*T*_FWHM_, and RCP) under the Magnetic Field Change of 0–50 kOe for the Gd–Pt–In and Some Other Reported Magnetocaloric Materials

materials	*T*_ord_ (K)	–Δ*S*_M_^max^ (J/kg K)	δ*T*_FWHM_ (K)	RCP (J/kg)	ref
Gd–Pt–In	67/64	6.0	63.5	368	this study
Gd_35_Ho_20_Al_25_Co_20_	66	9.76	74	574	^50^
Gd_3_Co_2_Ge_4_	31	7.9		246	^51^
Er_0.5_Gd_0.5_Al_2_	89	6.8	80	670	^52^
Er_41_Co_39_Gd_20_	64	9.66	42.3	702	^53^
GdNi_0.4_Al_1.6_	45	8.8	81	567	^54^
Gd–Co–Al	70	11	58	640	^55^
Gd_25_Dy_25_Co_25_Al_25_	61	9.1	72.9	663	^56^
Gd_50_Al_3_Co_20_/Gd_55_Al_20_ Co_25_	87	8.69		863	^57^

## 4 Conclusions

We synthesized a composite using the arc-melting method, and we analyzed its magnetic and magnetocaloric properties through magnetization and specific heat measurements. PXRD, combined with SEM/EDS data, confirmed that the Gd–Pt–In composite is primarily composed of a GdPtIn matrix phase and a dispersed GdPt phase.

The composite exhibits two closely spaced ferromagnetic transitions at 67 and 63.5 K. These dual transitions create a very large curve in both the −Δ*S*_M_ and Δ*T*_ad_ magnetocaloric parameters, leading to a broad temperature range with a significant MCE and a substantial relative cooling power. For a magnetic field change of 50 kOe, the maximum magnetic entropy change reaches 6 J/kg K, with a corresponding RCP value of 368 J/kg.

The excellent magnetocaloric properties of the Gd–Pt–In composite position it as a promising candidate for low-temperature magnetic refrigeration applications. Moreover, these findings offer valuable insights for the ongoing search for effective refrigerant materials, especially those characterized by multiple successive magnetic phase transitions.
